# Myostatin gene role in regulating traits of poultry species for potential industrial applications

**DOI:** 10.1186/s40104-024-01040-5

**Published:** 2024-06-03

**Authors:** Joonbum Lee, Dong-Hwan Kim, Kichoon Lee

**Affiliations:** Department of Animal Sciences, The Ohio State University, Columbus, OH 43210 USA

**Keywords:** Chicken, Economic traits, Myostatin, Quail

## Abstract

The myostatin (*MSTN*) gene is considered a potential genetic marker to improve economically important traits in livestock, since the discovery of its function using the *MSTN* knockout mice. The anti-myogenic function of the *MSTN* gene was further demonstrated in farm animal species with natural or induced mutations. In poultry species, myogenesis in cell culture was regulated by modulation of the *MSTN* gene. Also, different expression levels of the *MSTN* gene in poultry models with different muscle mass have been reported, indicating the conserved myogenic function of the *MSTN* gene between mammalian and avian species. Recent advances of CRISPR/Cas9-mediated genome editing techniques have led to development of genome-edited poultry species targeting the *MSTN* gene to clearly demonstrate its anti-myogenic function and further investigate other potential functions in poultry species. This review summarizes research conducted to understand the function of the *MSTN* gene in various poultry models from cells to whole organisms. Furthermore, the genome-edited poultry models targeting the *MSTN* gene are reviewed to integrate diverse effects of the *MSTN* gene on different traits of poultry species.

## Introduction

Poultry species provide a major source of dietary proteins from their meat and eggs. Poultry production is affected by various physiological processes which are regulated and influenced by many endogenous and exogenous factors. To benefit current poultry production and other economically important traits, genetic factors are considered potential major targets for regulating and improving most desired features in poultry species.

Nutritional and environmental factors are external aspects that can be readily modulated and thus have been actively studied in diverse poultry research. Genetic factors, however, are not easily accessible to investigate due to scarce research models, especially in avian species, despite their importance on regulation of economically important traits. Studies for genetic factors often require specific animal models to correlate their phenotypes with the genes of interest [1]. Improvement of analytic methods and tools revealed the association between specific traits and genetic variants and mutations such as single nucleotide polymorphisms in poultry species [2–4]. However, further experimental demonstrations are still required to conclude that the phenotypes are regulated by specific genes.

Researchers are currently using genome editing techniques as an innovative approach to artificially lead to phenotypic alterations in organisms. The emergence of the CRISPR/Cas9 system has revolutionized genome editing in almost all organisms, including avian species [5, 6]. By targeting specific genes of interest, genome-edited animal models show phenotypic changes caused by the modulation of the genes. These distinct phenotypes between the mutant and wild-type (WT) animals enable us to investigate the function of the gene directly and readily. Indeed, the function of the myostatin (*MSTN*) gene was first discovered in *MSTN* knockout mice by homologous recombination in embryonic stem cells and injection of the genome-edited cells into blastocysts [7], indicating the importance of genome editing methods in functional studies of genetic research.

Due to the increased muscle mass caused by the *MSTN* mutations in various farm animals [8–13], the *MSTN* gene has been considered a potential genetic marker to improve meat production in industry. Moreover, other benefits, such as decreased fat deposition [14–16] and improved bone quality [17, 18], also suggest immense potential of the *MSTN* gene in industry. Notably, generation of the genome-edited quail and chickens targeting the *MSTN* gene [19, 20] facilitates diverse research investigating the gene function in avian species. In this review, we discussed previously reported studies focusing on investigation of the *MSTN* gene function in different cells and poultry breeds across different species. In particular, functional studies using the *MSTN* mutant quail and chicken provide deeper insights into potential usage of the *MSTN* gene to improve various economic traits and well-being of poultry species.

## Myogenic function of the *MSTN* gene in poultry species

### Cell culture studies

The function of the *MSTN* gene has been investigated in several avian cell culture systems. When recombinant myostatin was treated into embryonic myoblasts, it inhibited proliferation and differentiation in chickens [21] and proliferation in turkeys [22]. The anti-myogenic effect of MSTN was also confirmed in breast muscle derived satellite cells from chicken and turkey after hatching [22, 23]. In addition, overexpression of the *MSTN* gene also inhibited proliferation and differentiation of the chicken embryonic myoblasts, resulting in reduction of the total myotube areas [24]. Moreover, inhibition of the *MSTN* gene expression by shRNA promoted proliferation of the duck embryonic myoblasts [25]. Also, knockout and overexpression of the *MSTN* gene in the quail myoblast cell line resulted in an increase and decrease of myotube formation, respectively [26, 27], indicating regulation of myogenesis by the *MSTN* gene.

### Different poultry breeds

As a top-down approach, different breeds of poultry species showing specific traits can be used to investigate whether the gene of interest plays a role in the specific traits of the breeds. Broilers are any chicken breeds that are intentionally selected and specifically raised for meat production [28], showing an excessive amount of muscle mass at their market ages, generally 6 weeks of age. Unexpectedly, the *MSTN* gene expression of broilers is not consistently lower than layers throughout pre- and post-hatching periods [29–31]. In one study, broilers showed increased MSTN expression at 2 weeks of age but decreased MSTN expression at 4 and 7 weeks of age [32]. Another study showed higher MSTN expression in broilers only at 5 days of age compared to layers [30]. Although broilers have higher body weight compared to layers since the hatching day [33], similar expression of the *MSTN* gene between broilers and layers throughout the embryonic stages, when myoblasts are formed, proliferated and differentiated [34], indicates that the *MSTN* gene might not be the major driving force of the different muscle mass between broilers and layers. Indeed, more than 50 years of genetic selection of broilers for better growth rate and body weight resulted in genetic variants between broilers and layers [35], suggesting compound effects of multiple genes for the higher muscle mass in broilers compared to layers.

However, the *MSTN* gene is still considered one of major genes regulating body weight and muscle mass in different poultry breeds. As a small-sized slow growing breed, Daweishan mini chickens showed higher expression of the *MSTN* gene compared to broilers [36], suggesting that the *MSTN* gene negatively regulates its body and muscle growth. Similarly, a quail breed selected for low body weight also showed higher MSTN expression compared to random bred control (RBC) quail at embryonic periods [37]. Additionally, a heavy weight quail line showed a decrease in the MSTN expression compared to RBC quail at pre- and early post-hatching periods [38]. The association between higher and lower MSTN expression with smaller and bigger body weight, respectively, in different poultry breeds indicates that the *MSTN* gene is one of main genes that regulate body weight and muscle mass in poultry species.

### Genome-engineered poultry models

Genome engineering method targeting the gene of interest is a direct approach to investigate the gene function on phenotypic changes of the animals. Currently, multiple genome-engineered poultry models targeting the *MSTN* gene have been generated via different methods (Table 1). Before the emergence of the CRISPR/Cas9 system, the function of the *MSTN* gene was investigated by overexpressing shRNA to inhibit its expression in transgenic chickens generated using a sperm-mediated gene transfer method [39, 40]. Although total dressing percentage and breast muscle percentage of the knockdown group were not different from the control group, body weight and leg muscle dressing percentage were increased in the knockdown chickens [40]. In another study, the MSTN–B form, a truncated peptide which inhibits the anti-myogenic function of MSTN–A form, was overexpressed in transgenic quail, using the lentivirus-mediated method [41]. Because expression of the MSTN–B form was specific in leg muscle of the transgenic quail, not throughout their body, transgenic quail showed increased muscle mass in their leg [41], suggesting improved muscle mass by inhibition of MSTN activity in poultry species.

**Table 1:** Currently reported whole-body genome-engineered avian models targeting the myostatin (*MSTN*) gene

Species	Method	Genome engineering approach	Target traits
Chicken	Sperm-mediated gene transfer	Overexpression of shRNA targeting the *MSTN* gene	Body weight [39, 40]; Muscle [40]
Quail	Lentivirus-mediated overexpression	Overexpression of MSTN–B form	Muscle [41]
Quail	Adenovirus-mediated genome editing	3 nucleotide deletion mutation by CRISPR/Cas9	Body weight, muscle and fat [19]; Feed efficiency and fat [42]; Bone size and quality [43, 44]; Egg production [45]; Eggshell quality [46]
Chicken	PGC-mediated genome editing	14 nucleotide deletion mutation & multiple indel mutations by CRISPR/Cas9	Body weight, muscle and fat [20]
Chicken	PGC-mediated genome editing	Base editing and multiple indel mutations by CRISPR/Cas9	Not applicable [6]
Duck	Adenovirus-mediated genome editing	1 nucleotide insertion mutation by CRISPR/Cas9	Not applicable [47]

As avian genome editing studies have been accelerated by the CRISPR/Cas9 system [5], genome-edited quail [19] and chickens [20] targeting the *MSTN* gene were generated using the adenovirus-mediated method and PGC-mediated method, respectively. The *MSTN* mutant quail showed significantly increased body weight compared to WT quail after 2 weeks of age in females and 3 weeks of age in males [19]. Increased number of muscle fibers, called hyperplasia, was shown in breast and leg muscles of the male and female mutant group compared to those of the WT group. In chickens, total body weight was not significantly increased by the *MSTN* mutation, although the mutant group showed a significantly higher growth rate compared to the WT group [20]. Nevertheless, leg and wing muscles, but not breast muscle, of the *MSTN* mutant chickens were heavier compared to those of the WT chickens [20]. Interestingly, muscle fiber hyperplasia was shown in leg and wing muscles of male mutant chickens, whereas female mutant chickens showed an increase in muscle fiber size only in the leg muscle [20]. These results suggest that the *MSTN* gene might not only act via different cellular mechanisms among muscle tissues at different locations but also presents sexual dimorphism in regulation of muscle mass in chickens.

In addition to the whole muscle mass, muscle fiber type I, but not fiber type II, was enlarged by the *MSTN* mutation in male chicken wing muscle [20]. Nonetheless, the number of type I fibers of the wing muscle in the *MSTN* mutant group was decreased [20]. Indeed, it has been suggested that one of the mechanisms of the *MSTN* gene on regulating muscle mass is by switching muscle fiber types, from slow oxidative type I and fast oxidative glycolytic type IIa to fast glycolytic type IIb [48]. Type II muscle fibers are responsible for an increase in muscle size, called hypertrophy, in the *MSTN* knockout mice and pigs [49, 50]. In breast muscles of duck and quail, which have both type IIa and IIb, IIb muscle fibers are larger in size and smaller in number than IIa fibers, and located on the outer edge of the muscle bundles [51, 52]. Therefore, the muscle fiber type transition from type IIa to IIb in the deep region of the breast muscle might partially support the increase in muscle mass of the *MSTN* mutant quail [52]. However, chicken breast muscle contains muscle fiber type IIb only [51], potentially limiting the effect of the *MSTN* mutation on regulating muscle fiber types and breast muscle mass. Because breast muscle is the largest muscle in chickens, the diminished effect of the *MSTN* mutation on body weight in chickens can be partially explained by the similar breast muscle weight between the *MSTN* mutant and WT chickens. Duck breast muscle is similar to quail breast muscle in terms of muscle fiber types [51]. Although functional studies have not yet been performed on the recently reported *MSTN* mutant duck model [47], it is expected that phenotypic effects would be similar to the results of the quail, but not the chicken.

## Additional traits examined in the genome-edited poultry species

### Fat deposition and feed intake

In addition to the increased muscle mass, decreased fat deposition is another major phenotypic change caused by the *MSTN* mutation in various farm animals [14, 15, 53, 54], suggesting an additional benefit of the *MSTN* mutation for production of leaner meat. Lean meat is not only favorable for consumers who are looking for healthier foods, but also beneficial to the producer by increasing meat yield. Excessive fat accumulation in broiler chickens is one of major problems in the modern broiler industry, because it is associated with meat yield, feed efficiency [55], and mortality [56]. In addition, excessive fat accumulation can also negatively affect laying performance and health in broiler breeder hens [57, 58].

Like in other *MSTN* mutant animals, fat deposition was reduced by the *MSTN* mutation in quail as shown in lower leg and abdominal fat percentages in both male and female quail [42], although leg and abdominal fat weight was significantly different only in female quail [19]. Decreased abdominal fat deposition was also shown in the *MSTN* knockout male chickens [20] while fat deposition of female chickens was not affected by the *MSTN* knockout [20]. Male chickens are bigger than female chickens, whereas female quail are bigger than male quail which could increase energy requirements for growing and maintaining their bigger bodies. When the body and muscle weight further increased by the *MSTN* mutation, it might redistribute energy expenditure more toward muscle accretion but away from fat deposition during the post-hatch growth. Along with the opposite sexual dimorphism of decreased fat deposition by *MSTN* mutation between quail and chickens, potential interspecies difference in various effects of the *MSTN* mutation among poultry species should be also investigated.

In broilers, the high fat line showed higher feed conversion ratio (FCR) compared to the lower fat line [55], indicating feed efficiency is affected by fat deposition in poultry species. As expected from the decreased fat deposition, the *MSTN* mutant quail showed lower FCR compared to WT quail in both male and female [42], confirming improvement of feed efficiency by the *MSTN* mutation. FCR was calculated by measuring feed intake (FI) and body weight gain (WG) during the growth period before sexual maturation. Although WG was higher in the mutant group compared to the WT group at all growth phases, FI was increased by the *MSTN* mutation at early- and mid-growth phases but not at late-growth phase. This prolonged WG without increased FI at late-growth phase resulted in decreased FCR at overall growth period in the *MSTN* mutant male and female quail [42]. Because feeding is a major part of production cost, improving feed efficiency by *MSTN* mutation could significantly reduce poultry production cost.

### Bone size and quality

Positive effect of the *MSTN* mutation on bone quality, demonstrated in *MSTN* knockout mice [17], can be another beneficial feature of the *MSTN* gene in the poultry industry, because poor bone qualities of both broilers and layers raise serious welfare and economic issues [59–61]. *MSTN* mutant pigs and rabbits also showed morphological changes in their rib and pelvic bones, respectively [62, 63], further supporting the potential role of the *MSTN* gene on bone formation. Because genetic factors are considered as major contributors to bone quality, along with nutritional and environmental factors, genetic approaches to improve traits related to bone qualities has been suggested as a potential solution [64]. In the *MSTN* mutant quail, bone size and quality were precisely analyzed by Micro-Computed Tomography scanning [43, 44]. Although there might be some indirect effect on bone size by increased body weight and muscle mass, significant increase in the size of tibia bones, including length, width, and cortical thickness, of both male and female quail by *MSTN* mutation supports the direct role of the *MSTN* gene on bone size in quail.

Other bone quality related parameters, such as bone mineral content, bone mineral density, bone volume, and bone breaking strength (BBS) were also higher in the *MSTN* mutant group compared to the WT group of adult male quail [43]. *MSTN* mutant female quail before sexual maturation also showed improvement in their bone quality related parameters [44]. However, the benefit of *MSTN* mutation on bone quality disappeared in female quail after sexual maturation, when most bone quality related parameters were similar between the two groups [44]. In fact, after the onset of egg laying, birds undergo dramatic changes to form a medullary cavity inside the long bone functioning as an accessible source of calcium for eggshell formation [65]. Notably, diaphyseal medullary and metaphyseal trabecular bones in *MSTN* mutant female quail had lower total surface value compared to WT female quail after sexual maturation. Because BBS of adult female quail between the 2 groups was similar, these negative values might be related to other physiological functions, such as calcium mobilization for eggshell formation, rather than bone strength.

### Egg production and eggshell quality

Fecund egg laying of poultry species makes them prosperous animals in the egg production industry. The effect of *MSTN* mutation on egg production traits was further confirmed in *MSTN* mutant quail [45], after an SNP in the *MSTN* gene, G2283A, was reported to be associated with body weight, age of first egg laying, and egg numbers [66]. The eggs of *MSTN* mutant quail were bigger and heavier compared to those of WT quail [45, 46]. Because the positive association between hens’ body weight and their egg size was reported in chickens [67, 68], heavier body weight of the *MSTN* mutant quail might have an indirect effect on bigger egg size in the mutant group compared to the WT group. Notably, egg weight was bigger in the *MSTN* mutant group than the WT group even relatively to their body weight [69], suggesting a potential direct effect on the egg weight by *MSTN* mutation in addition to the increased body weight of the mutant female quail. However, total egg production (egg weight (g) multiplied by produced egg numbers during the selected periods within the actively laying period) was similar between the two groups, because of lower number of eggs laid by the mutant group than WT group during the period [45]. Also, the onset of egg laying, another economic trait of layers, was delayed in the *MSTN* mutant female quail. Increased fat accumulation before sexual maturation in chickens and quail [70, 71] is required prior to initial egg laying. Lower fat deposition in the mutant female compared to the WT female [19] might be one of the contributors to this delayed onset of egg laying in the mutant group. Nevertheless, similar fertility and hatchability between the two groups indicates that egg quality and embryonic development are not negatively affected by *MSTN* mutation [45].

Like egg size, eggshell strength is also positively associated with avian body weight [72]. However, eggshell strength of the mutant eggs, analyzed by breaking strength, was lower compared to that of WT eggs, mainly due to decreased eggshell thickness [46]. Reduction in the thickness of the palisade layer in the mutant group played a major role in making the eggshell thinner and weaker than the WT group [46], because a palisade layer is the thickest and major calcified layer in the eggshell [73]. In addition, a decreased expression level of MSTN in the uterus, the specific region of avian oviduct for eggshell formation, in egg-laying hens compared to non-laying hens at the same age further supports a potential regulatory role of the *MSTN* gene in eggshell formation [74].

## Other considerations of the *MSTN* gene function in poultry species

### Alternative splicing isoforms

**Fig. 1:**
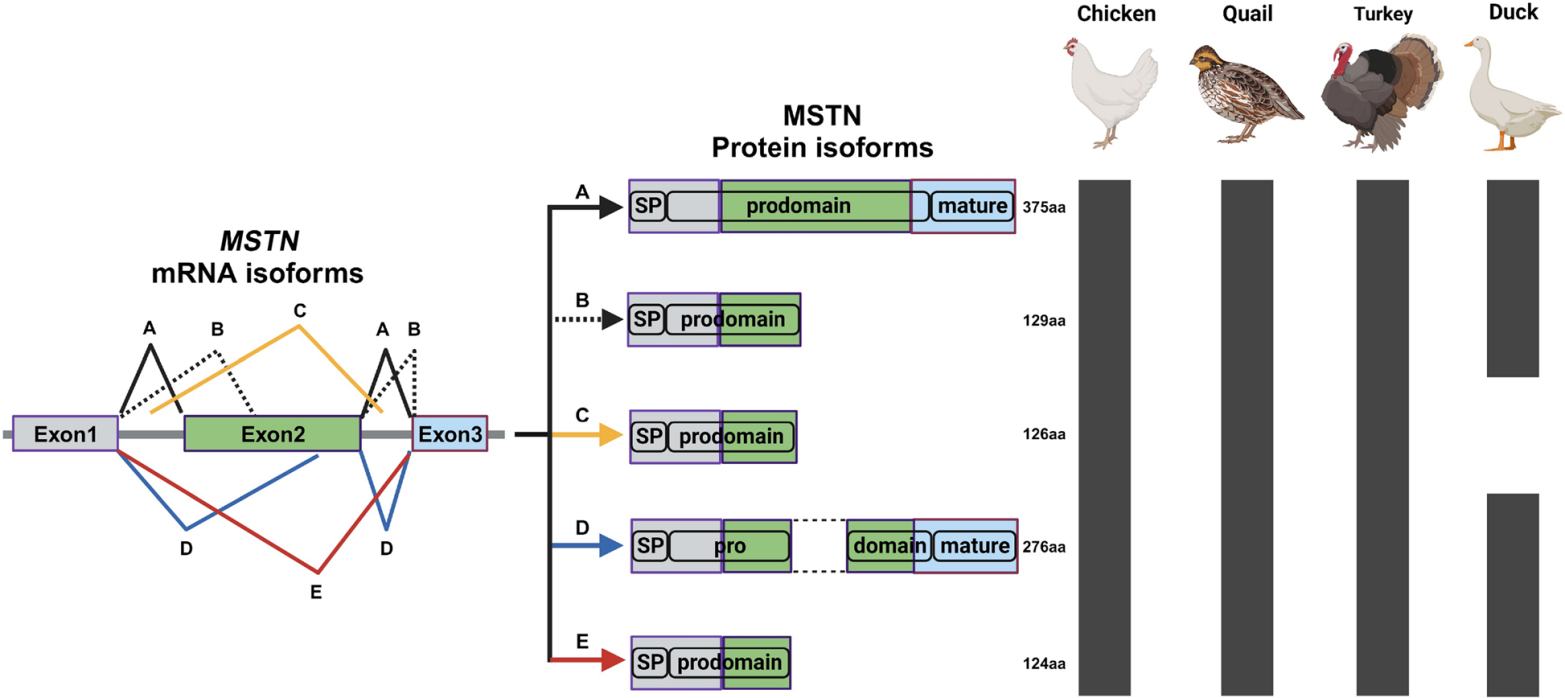
Schematic representation showing reported alternative splicing isoforms of the *MSTN* gene in major poultry species. Five isoforms including MSTN–A, B, C, D, and E forms have been identified in chickens, quail, and turkeys. In ducks, MSTN–A, B, D, and E forms are reported, but not MSTN–C form

Even though most of the research investigating expression of the *MSTN* gene reports the gene expression as a whole, 5 different isoforms of the MSTN have been reported in poultry species [27, 66] (Fig. 1). Among them, MSTN–A and MSTN–B forms are dominantly expressed in poultry species and have opposing functions on muscle growth [27]. MSTN–A is an intact form having the anti-myogenic effect on muscle growth as shown in reduced myotube length and diameter in quail myoblast cells after its overexpression [27]. On the contrary, overexpression of the MSTN–B form positively affected myogenesis of quail myoblast cells resulting in longer and thicker myotubes [27]. It was proposed that the MSTN–B form directly binds to the premature form of MSTN to inhibit the maturation process required for activation of MSTN [27]. In natural quail lines, the expression of the MSTN–B form was detectable after post-hatching d 28 [38]. The expression patterns of both forms correspond to the overall MSTN expression pattern of different lines of quail selected for their body weight [37, 38]. In chickens, however, both forms are detected even at embryonic ages and overall MSTN expression level does not represent the expression patterns of both forms [30]. Although MSTN expression as a whole was not different between broilers and layers at embryonic and post-hatching periods except for the early post-hatching day, the ratio of B form to A form was constantly higher in broilers compared to layers, except for the late embryonic day [30]. Higher expression of the B form in broilers compared to layers might suggest different genetic variation in broilers to inhibit MSTN activity and increase muscle mass. Thus, different expression patterns among MSTN isoforms can be another possible way to regulate muscle mass using the *MSTN* gene in poultry species.

### Environmental factors

Climate change is a real problem in our society and livestock industry [75, 76]. Global warming can increase environmental and heat stresses that need to be resolved for sustainable production [77, 78]. Appropriate temperature setting is an important factor for optimal growth in poultry species [79, 80], and expression of the *MSTN* gene is also affected by different temperature in chickens. Under constant heat stress (31 ± 1 °C) compared to thermoneutral control (21 ± 1 °C) during the growth period, it is reported that MSTN expression is elevated and growth performance is negatively affected in broilers at market age [81]. On the contrary, four-day-old chicken embryos exposed to hot temperatures (44 ± 0.5 °C) once for an hour decreased MSTN expression level immediately [82], altering the expression pattern of the *MSTN* gene during embryogenesis [83]. Interestingly, 1 week old chick exposed to 4 °C for a day increased breast muscle weight and decreased MSTN expression [84], suggesting a potential association between body temperature and expression of *MSTN* gene during embryonic and early post-hatching growth. In addition to the thermal stress, environmental stress coming from high stocking density needs to be managed for regulating MSTN expression properly. Under high stocking density conditions, broilers showed not only a decrease in average daily gain and breast muscle yield, but also an increase in the expression of the *MSTN* gene [85]. Therefore, indirect effects from various environmental stresses on avian growth performance through the MSTN expression should also be considered in an industrial setting.

## Conclusions

**Fig. 2:**
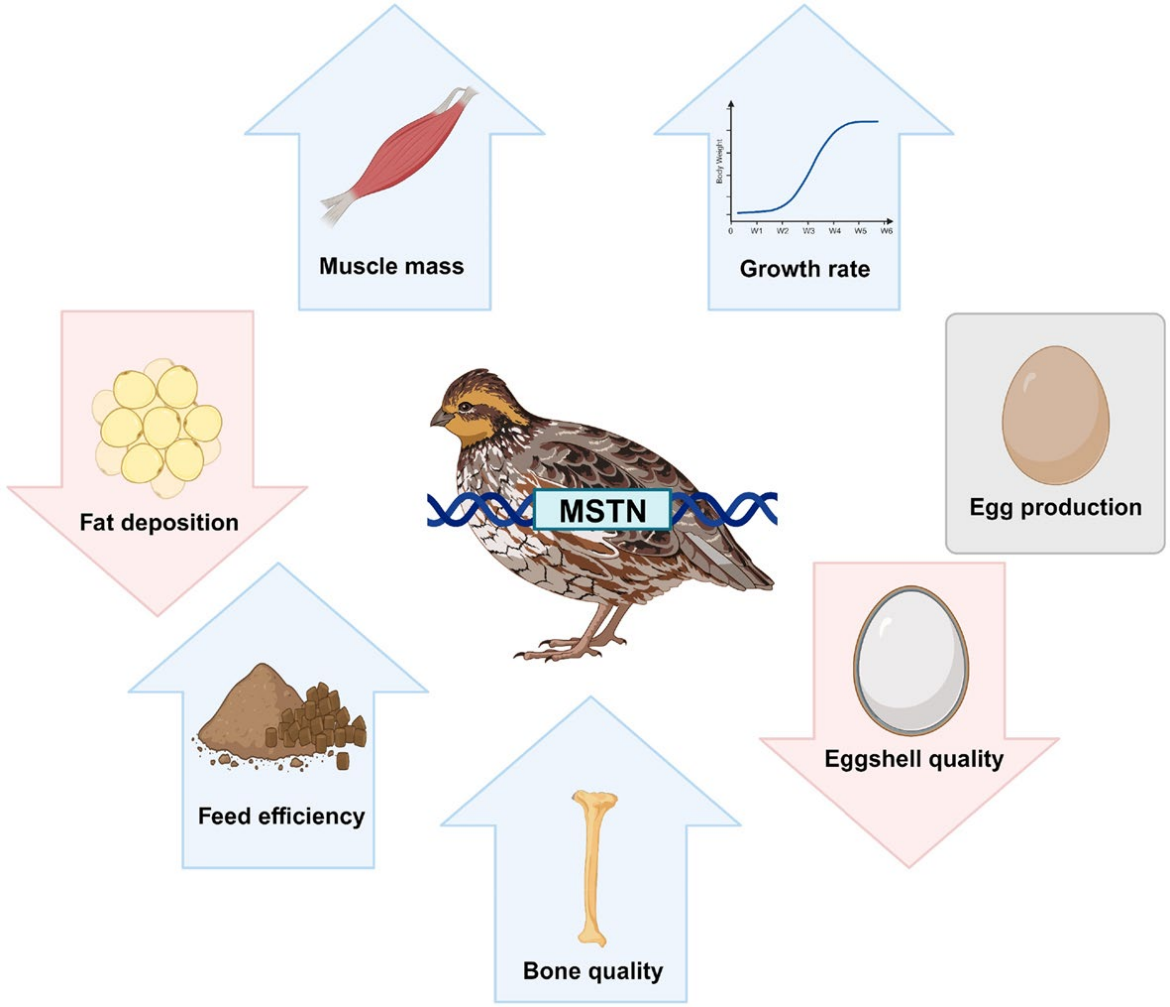
Illustrative representation of the effects of *MSTN* mutation on various traits in quail. *MSTN* mutant quail showed increased muscle mass and growth rate, decreased fat deposition, and improved bone quality, growth rate, and feed efficiency. However, *MSTN* mutant female quail did not show improvement in egg production and bone quality at egg laying period. In addition, eggshell quality was decreased by the *MSTN* mutation

Generation of genome-edited poultry models targeting the *MSTN* gene enabled researchers to investigate various functions of the *MSTN* gene in poultry species. In addition to confirmation of the reported effects of the *MSTN* mutation on muscle mass, fat deposition, feed efficiency, and bone quality in animals, novel discoveries on changed egg production traits and eggshell qualities indicate the importance of the *MSTN* mutant poultry models (Fig. 2). It is notable that most phenotypic changes caused by the *MSTN* mutation in quail and chickens are positively related to economic traits and health of meat producing poultry species. Also, an increase in muscle size of the *MSTN* mutant quail did not affect meat quality in terms of related traits, such as pH, redness, yellowness, and drip loss [52]. Because genome-edited farm animals have been lately approved for human consumption [86, 87], the *MSTN* gene can be seriously considered not only as a genetic marker but also for generation of a superior genome-edited line of poultry. Considering the high demand and importance of poultry meat in our society, comprehensive understanding of the *MSTN* gene in various traits of poultry species through genome-edited models and diverse research will greatly benefit not only poultry industry but also consumers.

## Data Availability

Not applicable.

## Abbreviations

BBS: Bone breaking strength
FCR: Feed conversion ratio
FI: Feed intake
MSTN: Myostatin
RBC: Random bred control
WG: Weight gain
WT: Wild-type
